# Association between HDL cholesterol with diabetic retinopathy in diabetic patients: a cross-sectional retrospective study

**DOI:** 10.1186/s12902-024-01599-0

**Published:** 2024-05-10

**Authors:** Wuping Xu, Xuedong Xu, Min Zhang, Chiping Sun

**Affiliations:** Department of Ophthalmology, The First People’s Hospital of Jiangyin District, Wuxi, Jiangsu 214400 People’s Republic of China

**Keywords:** High-density lipoprotein, Cholesterol, Diabetic retinopathy, NHANES

## Abstract

**Objective:**

Diabetic patients are often comorbid with dyslipidemia, however, the relationship between high-density lipoprotein cholesterol(HDL-C) and diabetic retinopathy (DR) in the adult diabetic population remains to be fully elucidated.The aim of this study is to evaluate the associations between HDL-C and DR in the United States adults with diabetes.

**Methods:**

A total of 1708 participants from the National Health and Nutrition Examination Survey (NHANES) 2005–2008 were enrolled in the present study. Fundus images of all study subjects were captured and evaluated using a digital camera and an ophthalmic digital imaging system, and the diagnosis of DR was made by the severity scale of the Early Treatment Diabetic Retinopathy Study (ETDRS).Roche Diagnostics were used to measure serum HDL-C concentration. The relationship of DR with HDL-C was investigated using multivariable logistic regression. The potential non-line correlation was explored with smooth curve fitting approach.

**Results:**

The fully-adjusted model showed that HDL-C positively correlated with DR(OR:1.69, 95%CI: 1.25–2.31).However, an inverted U-shaped association between them was observed by applying the smooth curve fitted method. The inflection point of HDL-C(1.99mmol/l) was calculated by utilizing the two-piecewise logistic regression model. In the subgroup analysis, the inverted U-shaped nonlinear correlation between HDL-C and DR was also found in female, Non-Hispanic White, and lower age groups.

**Conclusion:**

Our study revealed an inverted U-shaped positive relationship between HDL-C and DR.The findings may provide us with a more comprehensive understanding of the association between HDL-C and DR.

## Introduction

Diabetes, the most common metabolic disease all over the world, can lead to systemic microangiopathy and neuropathy with its chronic progression, eventually affecting the quality of life and increasing the burden on families [1, 2]. The global prevalence of diabetes is on the rise, with approximately 529 million individuals currently worldwide. In the United States, there are 3.4 million people with diabetes, constituting 10.5% of the total population; additionally, half of American adults either have prediabetes or have already developed diabetes [3].

Patients with diabetes are often complicated with dyslipidemia. As one of the most common indicators in blood lipid detection, high-density lipoprotein cholesterol (HDL-C) has also been found to be an effective biomarker for a variety of diabetic complications in recent years. Costacou et al. conducted a cross-sectional study and found that highly elevated HDL-C was associated with an increased risk of coronary heart disease in individuals with long-term type 1 diabetes [4].Another recent published literature also showed that HDL-C may play a role in the relationship of high hemoglobin in kidney function in diabetes [5].

Diabetic retinopathy (DR) is one of severe ocular complications of diabetes, leading to severe visual impairment [6]. It is now one of the leading causes for adult blindness worldwide.In an epidemiologic study for the adult population in the United States, there were about 30 to 40% of patients with diabetes at risk of retinopathy [7]. The main pathological progress of DR includes retinal capillary damage, retinal hemorrhage, retinal exudate, neovascularization of optic disc or retinal, and intraretinal microvascular abnormalities [8, 9]. Studies have found that controlling blood sugar and stabilizing retinal photocoagulation can effectively prevent the development of DR [10]. Given that diabetes complicated with DR is a long process, DR is a preventable disease. As the incidence of DR increases year by year, it is necessary to identify the risk factors related to DR to prevent it as early as possible. The established systemic risk factors for DR includes hyperglycemia, hypertension, and hyperlipidemia [11]. Besides, DR has been previously reported to be associated with some laboratory parameters, such as hyperuricemia, oxidative stress, inflammatory markers [12–14], microalbuminuria, serum creatinine (Cr) [15], and C-reactive protein (CRP) [16]. In addition, some previous findings have recorded that HDL-C is related to systemic vascular diseases, especially in people with diabetes [17, 18]. However, there are few studies focus on the association between DR and HDL-C, and the relationship is controversial. In the present study, we explored the association between DR and HDL-C in patients with diabetes using data based on a representative sample of patients with diabetes from NHANES database.We hypothesized that DR and HDL-C have a dose response relationship, which may promote the development of prevention strategies.

## Methods

### Study population

**Fig. 1:**
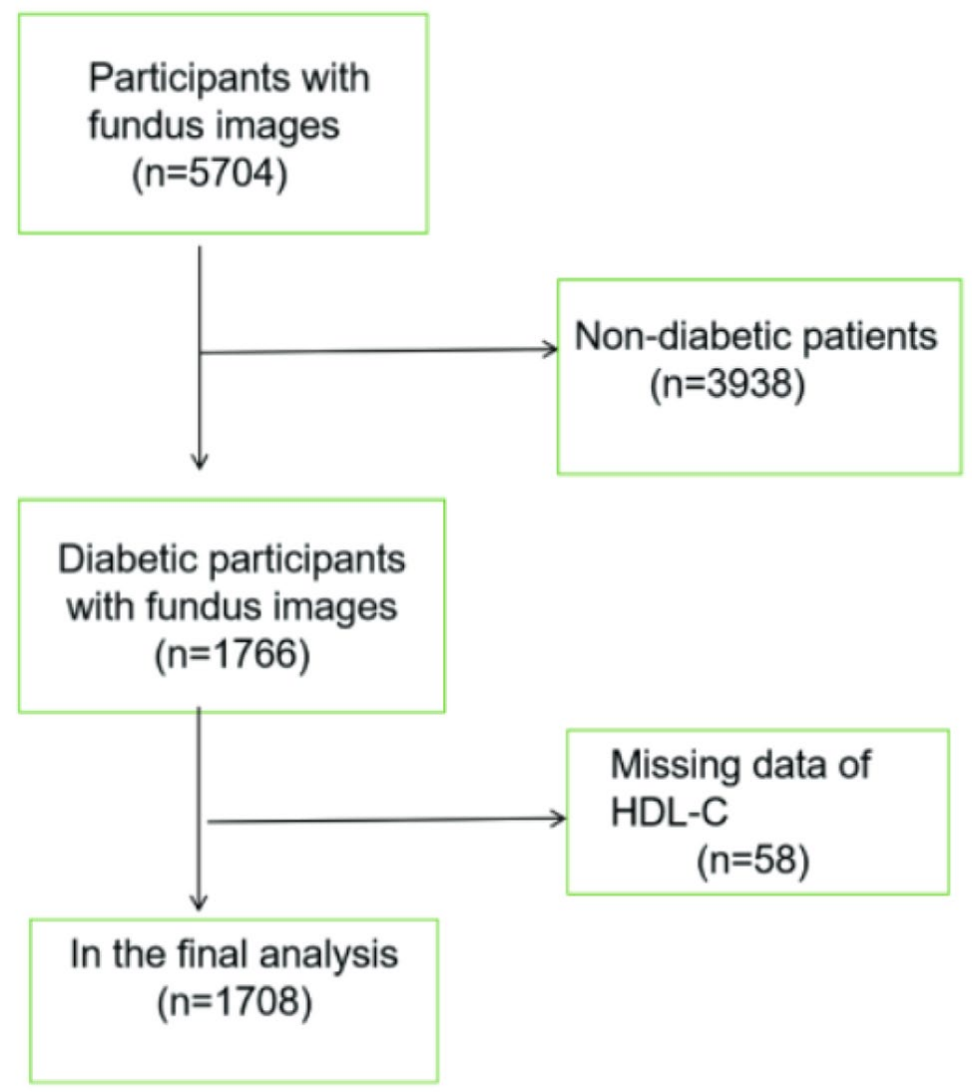
Flow chart of the participants enrolled in the present study

This cross-sectional study enrolled eligible participants from NHANES survey, which is a nationally representative study of the U.S. population. For more information about NHANES, visit its official website (www.cdc.gov/nchs/nhanes/). In this study, we pooled data from 2005 to 2008 for analysis, 5704 participants with available data of fundus images were considered eligible. Inclusion criteria for diabetes included: self-reported diabetes, use of insulin or anti-diabetes drugs, hemoglobin Alc level ≥ 6.5%, oral glucose tolerance test (OGTT) ≥ 11.1mmol/L, or serum glucose (Glu) ≥ 7.0mmol/L, meeting 2019 American Diabetes Association standards [19]. The exclusion criteria were as follows: (1) without diabetes (*n* = 3938) ;(2 )missing HDL data (*n* = 58). Data from the remaining 1708 patients with diabetes were included in our study (Fig. 1). The study was approved by the Institutional Review Board of the National Center for Health Statistics, and written informed consent was obtained from each participant [20].

### Variables

In the present study, DR and HDL-C were taken as Categorical and Continuous variables, respectively. Canon CR6-45NM ophthalmic digital imaging system and the Canon EOS 10D digital camera (Canon, Tokyo, Japan) were adopted for evaluating fundus photography. The fundus images of each eye were obtained through non-drug dilated pupils. At the Eye Epidemic Reading Center of the University of Wisconsin, Madison, the experienced photographer evaluated the fundus images using the modification of the Airlie House sorting system. The primary outcome variable, severity of retinopathy was recorded as no retinopathy and retinopathy, including mild non-proliferative retinopathy (NPR), moderate to severe NPR, and proliferative retinopathy [21]. The PEG-oxidase assay in Hitachi 717 and Hitachi 912 (Roche Diagnostics, Indianapolis, IN) or Roche Modular P chemistry OptiPlex 7070 SFF MLK analyzer (Diagnostic Roche) were used to measure serum HDL-C concentration.

The following data of continuous covariates were pulled out for inclusion in the study: age, duration of diabetes, Glu, HbA1c level, albuminuria, blood urea nitrogen (BUN), Cr, Serum globulin (Glb), systolic blood pressure (SBP), triglyceride (TG), C reactive protein (CRP). Moreover, the following categorical covariates were contained in our research: sex, race, overweight, arthritis, coronary heart disease (CHD), and stroke.

### Statistical analysis

Categorical variables were expressed as quantity (percentages). Continuous variables were shown as mean with standard deviation (SD) or percentages median (25th, 75th percentile). All the estimated weights of the NHANES sample were included in the calculation. The *P*-value was calculated using the weighted linear regression model for continuous variables and the chi-square test for categorical variables. The relationship between DR and HDL-C was explored by multivariate logistic regression and the smooth curve fittings after adjusting for other relevant clinical covariates. The inflection point was calculated by running a recursive algorithm. After non-linearity was detected, a weighted two-piecewise logistic regression model was further constructed by us.Statistical analyses were performed applying the EmpowerStats software (http://www.empowerstats.com) and R (version 4.1.1). *P* values < 0.05 were considered statistically significant.

## Results

**Fig. 2:**
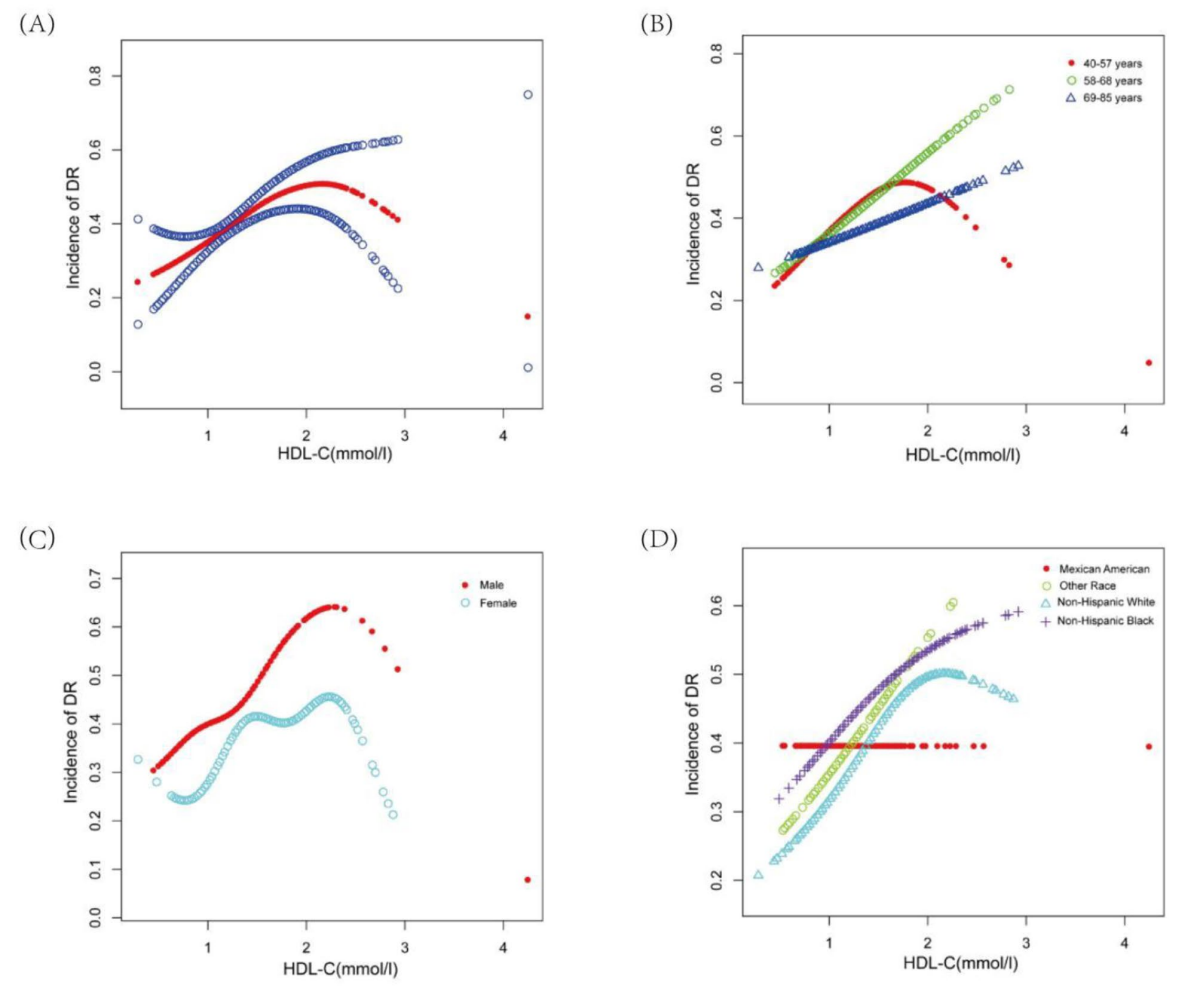
**(A)** The association between HDL-C and DR. Red line represents the smooth curve. Blue bands represent the 95% of confidence interval. Age, gender, race, duration of diabetes, Glu, HbA1c, BUN, albuminuria, Glb, Cr, SBP, TG, CRP, overweight, arthritis, CHD, stroke were adjusted. **(B)** Subgroup analysis stratified by age. Gender, race, duration of diabetes, Glu, HbA1c, BUN, albuminuria, Glb, Cr, SBP, TG, CRP, overweight, arthritis, CHD, stroke were adjusted. **(C)** Subgroup analysis stratified by gender. Age, race, duration of diabetes, Glu, HbA1c, BUN, albuminuria, Glb, Cr, SBP, TG, CRP, overweight, arthritis, CHD, stroke were adjusted. **(D)** Subgroup analysis stratified by race. Age, gender, duration of diabetes, Glu, HbA1c, BUN, albuminuria, Glb, Cr, SBP, TG, CRP, overweight, arthritis, CHD, stroke were adjusted

The detailed characteristics of 1708 participants enrolled in the present study are illustrated in Table 1. Age, duration of diabetes, Glu, HbA1c, SBP, TG, gender, race, overweight, arthritis are all significantly different among different groups of HDL-C (Tertiles, T1–T3). There was a positive association between HDL-C and DR in the unadjusted model [1.68 (1.31, 2.16)]. The similar outcomes were observed in model 2 (adjustment for age, gender, race) [1.93 (1.47, 2.54)] and model 3 (adjustment for age, gender, race, duration of diabetes, Glu, HbA1c, BUN, Albuminuria, Glb, Cr, SBP, TG, CRP, overweight, arthritis, CHD, stroke) [1.69 (1.25, 2.31)] (Table 2). Participants with higher HDL-C had a higher DR incidence than those with the lowest HDL-C levels in T1. The result of subgroup analyses stratified by age, gender, and race are shown in Table 3. Moreover, we performed weighted generalized additive models and smooth curve fittings to handle the nonlinear correlation and validate the outcomes. Smooth curve fitting is an important method to study the nonlinear relationship between risk factors and diseases, it has been adopted to investigate the nonlinear relationship between risk factors and the risk of various diseases in a large number of studies. The infliction point in a smooth curve is very helpful for public health policy makers to develop disease prevention strategies. We discovered an inverted U-shaped correlation between HDL-C and DR (Fig. 2A). In addition, in the subgroup analysis, we found an inverted U-shaped nonlinear relationship between HDL-C and DR in female, Non-Hispanic White, and lower age groups (Fig. 2B, C and D). However, in other subgroups, there was a positive linear correlation between HDL-C and DR. The results of the inflection points are indicated in Table 4.

**Table 1:** Participant characteristics

HDL-C (mmol/l) Tertiles	T1 (0.28–1.06)	T2 (1.09–1.37)	T3 (1.40–4.24)	*P* value
Age (years)	58.02 ± 11.37	59.48 ± 11.83	62.19 ± 11.42	< 0.0001
Duration of diabetes (years)	9.61 ± 4.92	10.30 ± 5.68	10.88 ± 5.37	0.0004
Glu(mmol/l)	7.87 ± 3.56	7.50 ± 3.13	6.88 ± 2.83	< 0.0001
HbA1c (%)	6.71 ± 1.53	6.50 ± 1.39	6.24 ± 1.33	< 0.0001
Albuminuria(mg/l)	14.30 (7.23–46.95)	12.50 (6.30–30.40)	11.80 (5.60-33.85)	0.3661
BUN(mmol/l)	5.32 ± 2.52	5.32 ± 2.35	5.37 ± 2.58	0.9204
Cr(µmol/L)	87.65 ± 34.06	86.61 ± 46.09	85.21 ± 40.89	0.6033
Glb(g/l)	29.51 ± 4.87	29.32 ± 4.32	29.18 ± 4.65	0.4894
SBP(mmHg)	129.62 ± 18.68	129.67 ± 19.57	132.92 ± 21.87	0.0060
TG(mmol/l)	2.38 ± 1.97	1.85 ± 0.68	1.63 ± 0.58	< 0.0001
CRP(mg/dl)	0.64 ± 0.83	0.56 ± 0.76	0.53 ± 1.22	0.1688
DR				< 0.0001
No	66.91	64.53	52.05	
Yes	33.09	35.47	47.95	
Gender				< 0.0001
Male	66.80	52.79	31.51	
Female	33.20	47.21	68.49	
Race (%)				0.002
Mexican American	8.69	7.04	4.94	
Other Race	8.53	10.04	8.19	
Non-Hispanic White	74.99	67.41	71.36	
Non-Hispanic Black	7.79	15.51	15.51	
Overweight (%)				< 0.0001
No	37.15	44.77	57.28	
Yes	62.85	55.23	42.72	
Arthritis (%)				0.0176
No	59.83	58.61	52.13	
Yes	40.17	41.39	47.87	
CHD (%)				0.5040
No	88.88	90.44	90.89	
Yes	11.12	9.56	9.11	
Stroke (%)				0.8308
No	91.47	91.55	90.64	
Yes	8.53	8.45	9.36	

Mean ± SD or Median (25th, 75th percentile) for continuous variables: P value was calculated by weighted linear regression model. % for categorical variables: P value was calculated by weighted chi-square test

**Table 2:** Association between HDL-C and DR

	Model 1 OR (95% CI)	Model 2 OR (95% CI)	Model 3 OR (95% CI)
HDL-C	1.68 (1.31, 2.16) < 0.0001	1.93 (1.47, 2.54) < 0.0001	1.69 (1.25, 2.31) 0.0008
HDL-C (Tertile)			
T1	1.0	1.0	1.0
T2	1.13 (0.89, 1.45) 0.3170	1.17 (0.91, 1.50) 0.2196	1.05 (0.80, 1.38) 0.7145
T3	1.64 (1.29, 2.09) < 0.0001	1.87 (1.45, 2.43) < 0.0001	1.69 (1.27, 2.26) 0.0004

Model 1, no covariates were adjusted

Model 2, age, gender, race were adjusted

Model 3, age, gender, race, duration of diabetes, Glu, HbA1c, BUN, Albuminuria, Glb, Cr, SBP, TG, CRP, overweight, arthritis, CHD, stroke were adjusted

**Table 3:** Association between HDL-C and DR, stratified by age, gender and race

	Model 1 OR (95% CI)	Model 2 OR (95% CI)	Model 3 OR (95% CI)
Stratified by age			
40–57 years	1.57 (0.99, 2.47) 0.0553	2.09 (1.25, 3.48) 0.0046	1.40 (0.79, 2.49) 0.2467
58–68 years	2.15 (1.39, 3.33) 0.0006	2.31(1.45, 3.68) 0.0004	2.35 (1.37, 4.04) 0.0020
69–85 years	1.47 (0.95, 2.27) 0.0808	1.47 (0.93, 2.32) 0.0999	1.29 (0.75, 2.20) 0.3561
Stratified by Gender			
male	2.22 (1.50, 3.30) < 0.0001	2.32 (1.54, 3.49) < 0.0001	2.07 (1.30, 3.30) 0.0021
female	1.73 (1.20, 2.48) 0.0032	1.65 (1.14, 2.39) 0.0085	1.34 (0.87, 2.07) 0.1793
Stratified by Race			
Mexican American	0.97 (0.52, 1.80) 0.9216	1.15 (0.61, 2.19) 0.6606	0.89 (0.43, 1.84) 0.7608
Other Race	1.69 (0.66, 4.37) 0.2770	2.66 (0.94, 7.57) 0.0661	5.23 (1.19, 23.04) 0.0286
Non-Hispanic White	2.01 (1.38, 2.92) 0.0002	2.41 (1.61, 3.60) < 0.0001	1.78 (1.12, 2.85) 0.0153
Non-Hispanic Black	1.54 (0.95, 2.50) 0.0766	1.68 (1.02, 2.76) 0.0431	1.68 (0.94, 2.98) 0.0781

Subgroup analyses stratified by age, gender and race, adjusted for duration of diabetes, Glu, HbA1c, BUN, Albuminuria, Glb, Cr, SBP, TG, CRP, overweight, arthritis, CHD, stroke

**Table 4:** Threshold effect analysis of HDL-C and DR by using piecewise logistic regression

	Adjusted OR (95% CI), *p* value
Total participant	
HDL-C < 1.99(mmol/l)	2.28 (1.57, 3.32) < 0.0001
HDL-C > 1.99(mmol/l)	0.36 (0.11, 1.17) 0.0885
Female	
HDL-C < 1.4(mmol/l)	3.39 (1.29, 8.92) 0.0135
HDL-C > 1.4(mmol/l)	0.78 (0.40, 1.52) 0.4661
40–57 years	
HDL-C < 1.91(mmol/l)	2.99 (1.42, 6.30) 0.0038
HDL-C > 1.91(mmol/l)	0.07 (0.00, 1.07) 0.0557
Non-Hispanic White	
HDL-C < 1.97(mmol/l)	2.52 (1.42, 4.45) 0.0015
HDL-C > 1.97(mmol/l)	0.26 (0.04, 1.78) 0.1704

## Discussion

The overall purpose of this study was to explore the association between DR and HDL-C in a nationally representative sample of NHANES 2005–2008. Hyperlipidemia has been widely acknowledged as a significant risk factor for the development of DR, particularly in relation to the formation of hard exudates. This association may arise from impaired lipid clearance within the diabetic retina, leading to increased non-enzymatic oxidation and glycosylation, activation of inflammatory mediators, and subsequent vascular hyperpermeability and disruption of the blood-retinal barrier in DR [22]. Meanwhile, previous studies on dietary interventions aimed at lowering lipids and fibrate therapy have demonstrated regression of retinal hard exudates, while a diet rich in polyunsaturated fatty acids has shown promising results in protecting against retinopathy [23]. HDL-C is a compound of several triglycerides, lipoproteins, and cholesterol. Previous studies have demonstrated that HDL-C was involved in numerous vascular pathologic mechanisms, most notably the vascular cell migration and proliferation [24]. Higher HDL-C concentration have abnormal and harmful effects on blood vessels and are related to vascular diseases like coronary heart disease, kidney diseases, and diabetes [25–28]. Nevertheless, the relationship between HDL-C and DR remains unclear. Some studies indicated that no significant relationship between DR and HDL-C, Morton et al. explored the association of baseline HDL-C with DR and found that HDL-C was not related to DR or any other retinopathy [29–31]. However, some literatures reported that HDL-C is correlated with DR, Sasso et al. [32, 33] found a remarkable positive correlation for the severity of DR with HDL-C levels’ rise. On the contrary, Popescu et al. [34, 35] reported that HDL-C was negatively related to the incidence of DR. The reasons for heterogeneity among previous studies may be related to discrepancies in research design, study sample, and control of confounding variables.

The most significant advantage of our study is that it contains a representative sample of a multi-racial population, which has a good universality of all American people. Moreover, the main discovery of our study is that the correlation between DR and HDL-C was in an inverted U-shaped curve pattern. The incidence of DR increased with HDL-C up to the turning point(1.99mmol/l). Subgroup analysis was also performed according to the STROBE guides to delineate the data in detail [36]. In subgroup analysis, the inverted U-shaped relationship between HDL-C and DR was also observed in female, Non-Hispanic White, and lower age groups. The thresholds of the subgroups are obtained, respectively. Differences in genetic risk factors, lifestyle habits and other factors may provide possible explanations for the special relationship among female, Non-Hispanic White, and lower age groups. Further large prospective studies are needed to elucidate the relationship between DR and HDL-C in these population groups. These findings suggest potentially optimal levels of HDL-C for DR, providing valuable insights for clinicians to enhance their understanding of the relationship between DR and HDL-C. HDL-C may be a potentially modifiable danger factor for DR. Measuring HDL-C levels may provide prediction and screening tools for DR patients, as well as to avoid an overcorrection of HDL-C among patients with DR. Therefore, we need more evidence to support this.

However, some limitations should also be clarified. First, considering our study is a cross-sectional study, ruling out any causal inference. Second, though we adjusted several important potential confounding factors, other confounders including body mass index, poor blood glucose control, dialysis, the incidence of other microvascular diseases, treatment of hypertensive and hyperlipidemia, dietary habits, nutritional uptake may also affect the result. More prospective studies with more comprehensive covariates are necessary to confirm this conclusion.

## Conclusions

A positive association was found between HDL-C and DR, and the correlation was in an inverted U-shaped pattern. Patients with higher HDL-C had a higher risk of DR, the inflection point was calculated as 1.99mmol/l. More attention should be paid to HDL-C in patients with diabetes to better prevent and treat DR.

## Data Availability

The survey data are publicly available on the internet for data users and researchers throughout the world (www.cdc.gov/nchs/nhanes/).

## Abbreviations

HDL-C: High-density lipoprotein cholesterol
DR: Diabetic retinopathy
NHANES: National Health and Nutrition Examination Survey: Glu: Serum glucose
HbA1c: Hemoglobin Alc
BUN: Blood urea nitrogen
Cr: Creatinine
Glb: Serum globulin
SBP: Systolic blood pressure
TG: Triglyceride
CRP: C reactive protein
CHD: Coronary heart disease
